# TLR2-mediated activation of innate responses in the upper airways confers antiviral protection of the lungs

**DOI:** 10.1172/jci.insight.140267

**Published:** 2021-03-08

**Authors:** Georgia Deliyannis, Chinn Yi Wong, Hayley A. McQuilten, Annabell Bachem, Michele Clarke, Xiaoxiao Jia, Kylie Horrocks, Weiguang Zeng, Jason Girkin, Nichollas E. Scott, Sarah L. Londrigan, Patrick C. Reading, Nathan W. Bartlett, Katherine Kedzierska, Lorena E. Brown, Francesca Mercuri, Christophe Demaison, David C. Jackson, Brendon Y. Chua

**Affiliations:** 1 Department of Microbiology and Immunology, the University of Melbourne, the Peter Doherty Institute for Infection and Immunity, Melbourne, Victoria, Australia.; 2 Viral Immunology and Respiratory Disease group, School of Biomedical Science and Pharmacy, Faculty of Health and Medicine, University of Newcastle, Newcastle, Australia.; 3 Priority Research Centre for Healthy Lungs, University of Newcastle and Hunter Medical Research Institute, Newcastle, Australia.; 4 WHO Collaborating Centre for Reference and Research on Influenza, the Peter Doherty Institute for Infection and Immunity, Melbourne, Victoria, Australia.; 5 Ena Respiratory, Melbourne, Victoria, Australia.

**Keywords:** Infectious disease, Therapeutics, Influenza, Innate immunity

## Abstract

The impact of respiratory virus infections on global health is felt not just during a pandemic, but endemic seasonal infections pose an equal and ongoing risk of severe disease. Moreover, vaccines and antiviral drugs are not always effective or available for many respiratory viruses. We investigated how induction of effective and appropriate antigen-independent innate immunity in the upper airways can prevent the spread of respiratory virus infection to the vulnerable lower airways. Activation of TLR2, when restricted to the nasal turbinates, resulted in prompt induction of innate immune–driven antiviral responses through action of cytokines, chemokines, and cellular activity in the upper but not the lower airways. We have defined how nasal epithelial cells and recruitment of macrophages work in concert and play pivotal roles to limit progression of influenza virus to the lungs and sustain protection for up to 7 days. These results reveal underlying mechanisms of how control of viral infection in the upper airways can occur and support the implementation of strategies that can activate TLR2 in nasal passages to provide rapid protection, especially for at-risk populations, against severe respiratory infection when vaccines and antiviral drugs are not always effective or available.

## Introduction

The onslaught of respiratory pathogens is felt not only during the emergence of novel viruses, such as the pandemic H1N1 influenza virus of 2009, SARS coronavirus, Middle East respiratory syndrome coronavirus, and SARS coronavirus 2 (the causative agent of COVID-19), but endemic seasonal virus infections also pose an equal and ongoing risk of severe disease and death for many individuals. Even when vaccines are in widespread use against viruses, for example influenza, their effectiveness can vary markedly (1) and protection cannot be guaranteed. Similarly, antiviral drugs are not available for all viruses; some that are effective against influenza virus can be used prophylactically (2), but their window of protection is short and ongoing use is not recommended because of the potential for the selection of drug-resistant variants (3). Furthermore, because most antiviral agents are specific to a particular virus family or genus, they may not be appropriate for unrelated endemic or emerging viruses. Clearly, there remains an unmet need for broadly active antiviral prophylactic therapies to prevent virus-induced respiratory complications, especially in highly susceptible individuals.

Effectors of innate immunity can be immediate and of sufficient vigor to limit infection. Although nonspecific in the sense that they lack the specificity of adaptive immune responses, innate immune responses can be specifically initiated by activation of TLRs, which exhibit a comprehensive range of recognition capabilities, from signatures of bacterial cell wall components to the RNA and DNA of viruses (4). These pathogenic signatures can alert the host cell to immediately mobilize innate effector mechanisms to combat infection processes, making them prime targets for inducing antiviral responses (5).

In this study, we examine how triggering innate immunity in the upper respiratory tract, an initial site of respiratory virus replication, prevents progression of influenza A virus (IAV) to the lower airways. Influenza virus primarily infects respiratory epithelial cells, beginning with those first encountered in the upper respiratory tract (URT) (6). Alerting epithelial cells occurs through recognition of the viral genome and viral proteins by TLRs, RIG-I–like receptors (RLRs), and NLRs (7, 8). Upon sensing viral invasion, epithelial cells can produce a range of antiviral mediators (9–11) as well as cytokines that promote recruitment of innate immune cells including macrophages, neutrophils, and NK cells to exert effector functions (12, 13). Engagement of the innate immune system prior to viral infection could therefore provide rapid prophylaxis by taking advantage of the multiple mechanisms inherent in innate host defenses.

Synthetic agonists of the viral DNA/RNA-recognizing TLR molecules, TLR3, TLR7/8, and TLR9, in particular, have been found to boost protective innate immune responses against a range of respiratory viruses (14–16). Their success in the clinic, however, could be limited mostly because of short duration of benefit or induction of adverse effects related to activation of the TNF-α and type 1 IFN pathways (17). In our study, we triggered the innate immune system through TLR2 only, which recognizes the S-[2,3-bis(palmitoyloxy)propyl] cysteine (Pam_2_Cys) moiety of bacterial lipopeptides and is present on the surfaces of epithelial cells and cells of the immune system. We (18–21) and others (14, 22) have previously studied the stimulatory effects of TLR2 agonists i.n. delivered into the respiratory tract, including the lower airways, to induce protective immunity against bacterial and viral infections.

We have now engineered Pam_2_Cys as an immunomodulatory molecule (INNA-X) and here describe an animal model that allows examination of its effects when localized to the URT rather than contacting the lower airways. We demonstrated that targeted delivery of INNA-X to the URT resulted in the rapid induction of a tissue-localized innate immune response characterized by the upregulation of genes associated with, and accompanied by, production of cytokines and the infiltration of innate immune cells. Administration of INNA-X to the URT before exposure to influenza virus reduced the viral burden in nasal epithelia and limited viral progression to the lungs. We also demonstrated that epithelial cells and macrophages work in concert and play pivotal roles in the antiviral defenses. These findings indicate a clear application for use of INNA-X as an immunomodulator to mobilize rapid protection against respiratory tract infection. Because the molecule targets the host and not the pathogen, the method of control is not likely to cause drug resistance and could be effective against currently circulating and emerging respiratory viruses, including those that can cause and/or exacerbate respiratory complications (23).

## Results

### Delivery of influenza virus and INNA-X to the URT.

**Figure 1:**
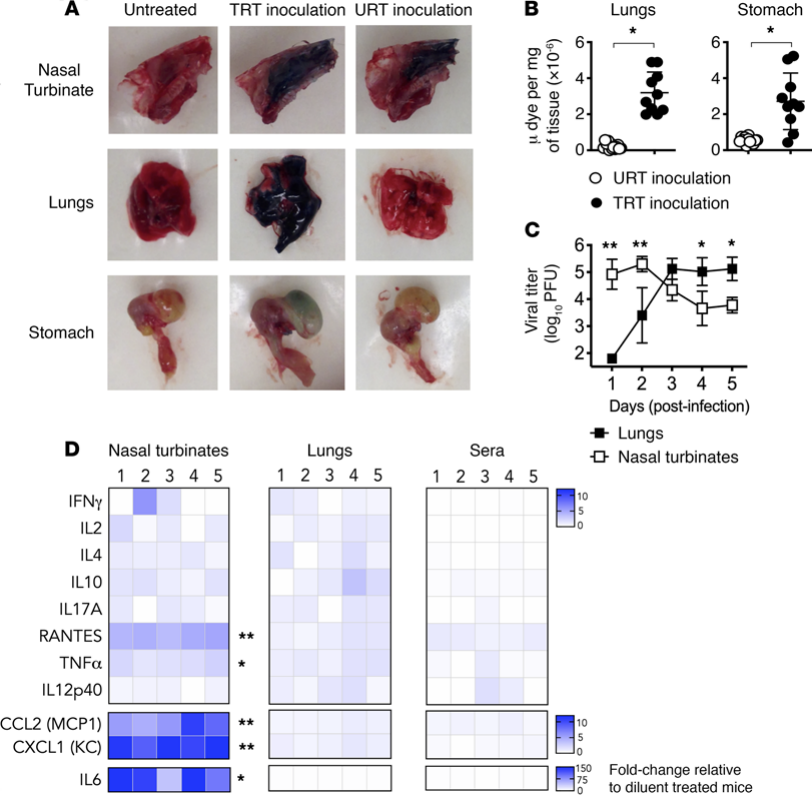
Distribution of inocula after administration to the URT or TRT. (**A**) Mice (*n* = 10/group) were i.n. inoculated with 0.125% Evans blue dye in PBS (10 μL URT inoculation or 50 μL TRT inoculation). Representative images of nasal turbinates (NT), lungs, and stomach dissected 2 minutes later are shown in comparison with an untreated control. (**B**) Supernatants from homogenized lungs and stomach were treated with trichloroacetic acid with absorbance measured at 620 nm. Concentrations of dye were interpolated from a standard curve. (**C**) Mice (*n* = 5/group) were infected with 500 PFU of Udorn IAV in 10 μL. Viral titers in NT and lungs were determined in an MDCK plaque assay. (**D**) The immunostimulatory activity of URT-inoculated INNA-X (5 nmol) was determined in mice (*n* = 5/group) by measuring cytokine levels by multiplex bead array 1 day after treatment. Statistical analysis was performed using a (**B**) Welch *t* test, (**C**) 2-way ANOVA with a Bonferroni post hoc test (relative to diluent-treated mice) and (**D**) multiple-comparison Holm-Sidak *t* test.*P < 0.01, **P < 0.001.

To model as closely as possible physiological infection of mice with influenza virus and to anatomically restrict treatment with INNA-X, we examined the distribution of i.n. delivered inocula throughout the respiratory tract. Inocula can be delivered to the total respiratory tract (TRT) or restricted to the URT, including nasal passages, by varying the volume administered (24, 25). Inoculation of Evans blue dye in a volume of 50 μL resulted in staining of the entire respiratory tract, including the nasal turbinates and lungs (Figure 1A). In contrast, administration of 10 μL restricted dye to the nasal turbinates. With the lesser volume, little or no dye was detected in the lungs and stomach, indicating restriction to the URT (Figure 1B). Similar results to those obtained with 10 μL were observed using volumes up to 15 μL (Supplemental Figure 1; supplemental material available online with this article; https://doi.org/10.1172/jci.insight.140267DS1).

To determine whether deposition of a small volume of viral inoculum in the nares is followed by sequential progression of virus throughout the respiratory tract, groups of mice were challenged with Udorn IAV and viral titers in nasal turbinates and lungs measured daily for 5 consecutive days after infection. Virus was detected in the nasal turbinates within 1 day of viral challenge with titers up to 1000-fold greater than the infectious dose administered (Figure 1C). No virus was detected in lungs at this time point, indicating that infection was confined to the URT. Progression of virus from the nose to the lungs was evident from day 2 after the challenge onward and reached a plateau after 3–5 days, concurrent with a decrease in viral titers in the nasal turbinates. We also found that restriction of INNA-X delivery to the URT resulted in containment of cytokine responses to the site of administration (Figure 1D). All subsequent experiments used small volumes of inocula with which to treat or infect mice.

### Treatment of the URT with INNA-X limits progression of influenza infection to the lungs.

**Figure 2:**
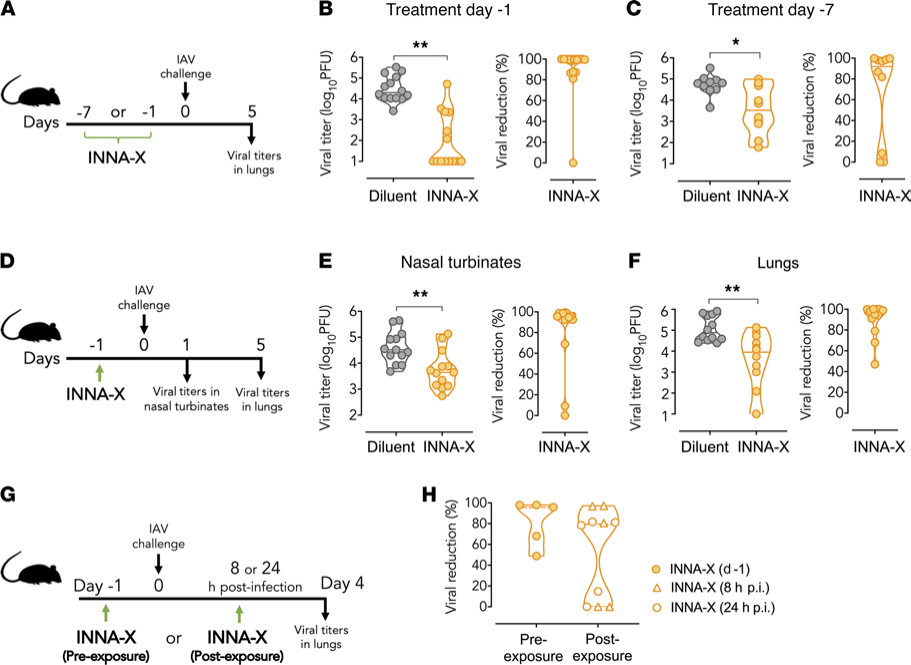
Inhibition of viral replication in the respiratory tract after administration of INNA-X to the URT. (**A**) Mice (*n* = 5 or 7/group) were inoculated with 5 nmol of INNA-X prior to challenge with 500 PFU of Udorn IAV. Efficacy of treatment (**B**) 1 day or (**C**) 7 days prior to viral challenge was determined by measuring lung viral titers 5 days after infection. The percentage reduction in viral load in each mouse is shown relative to the average viral titer in similarly challenged diluent-treated mice. Results (**B** and **C**) are pooled from 2 separate experiments. (**D**) Mice were inoculated with 1 nmol of INNA-X and challenged 1 day later. Viral titers in (**E**) nasal turbinates or (**F**) lungs (*n* = 7/group) were measured at 1 or 5 days, respectively, after infection. (**G**) Therapeutic efficacy of treatment with INNA-X was examined by inoculating mice with INNA-X 8 or 24 hours after IAV challenge (postexposure) in comparison to treatment 1 day prior to challenge (preexposure). (**H**) Reduction in lung viral titers is relative to similarly infected mice treated with diluent at each time point. Statistical analysis was performed using a (**B**, **E**, and **F**) Mann-Whitney or (**C**) Welch *t* test. *P < 0.05, **P < 0.01.

Application of INNA-X to the nasal turbinates 1 or 7 days before delivery of virus to the URT (Figure 2A) significantly reduced the amount of virus recovered from the lower respiratory tract. Lung viral loads, determined at the height of pulmonary viral replication (5 days after infection), were significantly (>80%) reduced in the majority of mice pretreated with INNA-X at either 1 day (Figure 2B) or 7 days (Figure 2C) prior to viral challenge. Viral loads were also significantly reduced in the nasal turbinates, as demonstrated using 5-fold less INNA-X (1 nmol) delivered 1 day prior to viral challenge and measured 1 day after infection (Figure 2, D and E). Similar reductions in lung viral loads were observed 5 days after viral challenge (Figure 2F). These results demonstrated that INNA-X conferred a rapid protective effect at the site of delivery in the URT and limited viral progression from that site, affording protection of the lower respiratory tract. In addition, the therapeutic efficacy of INNA-X treatment was also demonstrated in mice that were treated within 24 hours after exposure to influenza virus (Figure 2G), resulting in more than 80% reduction in lung viral titers in 60% of mice (Figure 2H).

### INNA-X induces rapid innate immune responses in nasal turbinates.

**Figure 3:**
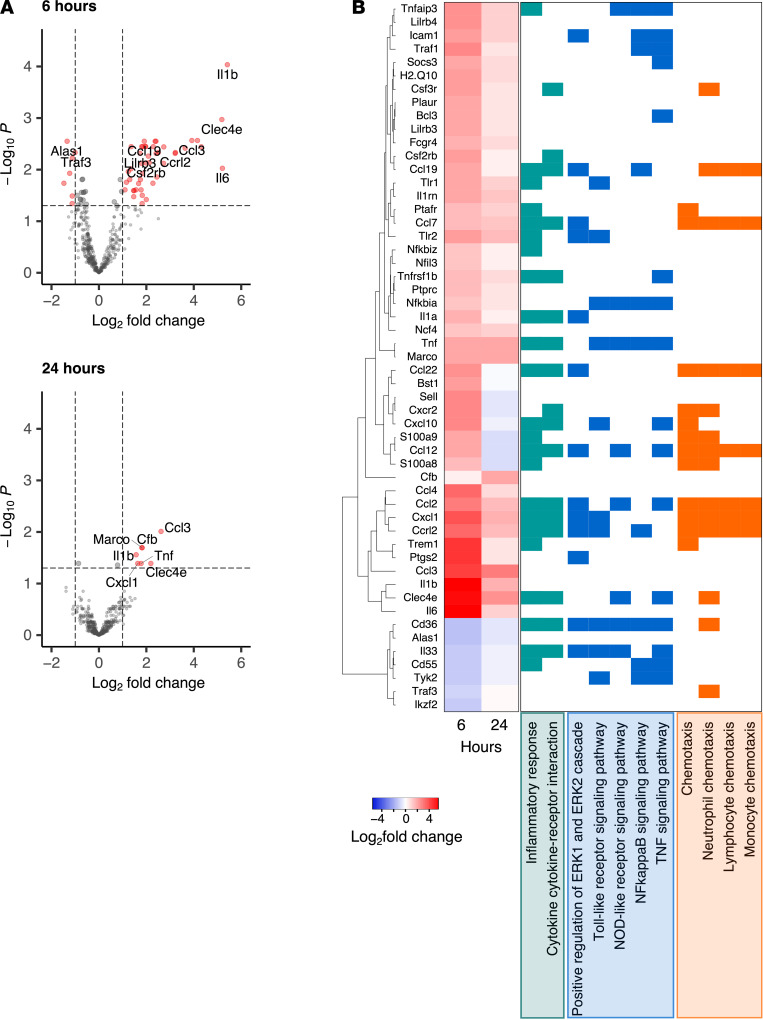
Gene expression changes induced by treatment with INNA-X. RNA was isolated from nasal turbinates 6 hours and 24 hours after administration of 1 nmol of INNA-X or diluent to the URT (*n* = 5/group). RNA was analyzed by NanoString nCounter assay against a panel of 547 immune genes. (**A**) Volcano plots showing log_2_ fold change and –log_10_ FDR. (**B**) Heatmap showing log_2_ fold change of differentially expressed genes (log_2_ fold change ≥ 1 or ≤ –1 and FDR < 0.05 at each time point) and clustered by expression. Genes with related functions, categorized by gene ontology terms, are highlighted in green (inflammatory processes), blue (danger sensing/signal transduction), and orange (chemotaxis).

Because INNA-X–mediated antiviral activity occurred in the URT within 1 day of inoculation, we investigated whether the effects of treatment were mediated by induction of local innate immune responses. Total RNA was harvested from nasal turbinate tissue 6 and 24 hours after treatment with INNA-X and hybridized to the nCounter mouse immunology panel for analysis of appropriate gene expression. Compared with animals treated with diluent vehicle, a total of 54 genes were differentially expressed, with more than 85% of genes upregulated within 6 hours of treatment (Figure 3A). Upregulated genes included those encoding CXCL1 (KC), CCL2 (MCP-1), CCL3 (MIP-1α), CCL4 (MIP-1β), and the cytokines TNF-α, IL-1α, and IL-1β that are associated with antiviral responses (Figure 3B). These genes, together with a significant number of others, are also typically associated with activation, chemotaxis, and function of immune cells. A smaller number of genes were upregulated after 24 hours (Figure 3A). These were largely representative of a subset of those detected at 6 hours after treatment but were expressed at lower levels, indicating that these genes were rapidly activated after treatment and downregulated shortly thereafter.

**Figure 4:**
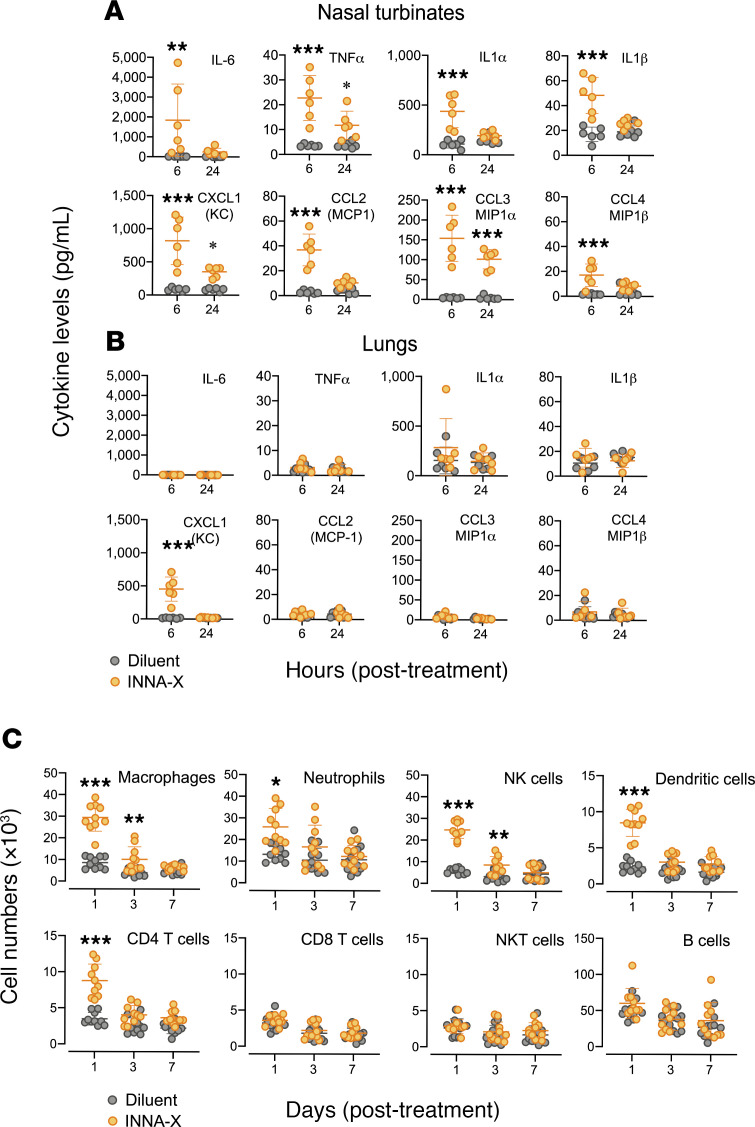
Cytokine levels and cell populations in the respiratory tract of mice after treatment with INNA-X. Mice (*n* = 3/group) were inoculated with 1 nmol of INNA-X or diluent only and 1 day later cytokine levels in (**A**) nasal turbinates and (**B**) lungs were analyzed in a multiplex bead array assay. (**C**) Nasal turbinates were harvested 1, 3, and 7 days after treatment (*n* = 5/group) and single-cell suspensions stained with fluorochrome-conjugated antibodies of various specificities and analyzed by flow cytometry. All results are pooled from 2 separate experiments. Statistical analysis was performed by 2-way ANOVA with a Bonferroni post hoc test. *P < 0.05, **P < 0.01, ***P < 0.001.

To determine the presence of gene products encoded by the upregulated genes in nasal turbinates and in particular IL-6, TNF-α, IL-1α, and IL-1β, as well as CXCL1, CCL2, CCL3, and CCL4, we determined their concentrations after treatment. Significantly higher levels of cytokines were detected 6 hours after exposure to INNA-X compared with treatment with diluent (Figure 4A). Whereas concentrations of IL-6, IL-1α, IL-1β, CCL2, and CCL4 returned to baseline levels 24 hours after treatment, TNF-α, CXCL1, and CCL3 remained significantly elevated, albeit at lower levels than those determined at 6 hours, corresponding to the downregulation of their respective genes. Notably, with the exception of CXCL1, there were no significant differences in levels of cytokines detected in the lungs between each treatment group at either time point (Figure 4B). In fact, cytokine levels in the lungs of mice remained largely unchanged over the course of 48 hours after INNA-X treatment (Supplemental Figure 2A), confirming that the direct action of INNA-X, when i.n. delivered and in a small volume, was localized to the URT.

The transient increase of cytokines in nasal turbinates of mice treated with INNA-X is also consistent with their function in recruiting immune cells. Phenotyping of cell populations in the nasal turbinates 1 day after treatment revealed an increased number of macrophages, neutrophils, NK cells, and DCs (Figure 4C). Additionally, CD4^+^ T cells were also increased early after treatment compared with animals that received diluent (Figure 4C). Although levels of neutrophils, DCs, and CD4^+^ T cells returned to baseline levels within 3 days, elevated numbers of interstitial macrophages and NK cells were still present at this time but waned over the next 4 days. In contrast, there were no significant changes in the numbers of CD8^+^ T cells, NKT cells, and B cells in animals that received INNA-X compared with diluent treatment at any of the times examined. In contrast, no significant differences in frequencies of these cell populations were observed in the lung (Supplemental Figure 2B). Altogether, these results demonstrated that exposure of the nasal turbinates to INNA-X resulted in rapid local expression of genes and their products, which led to the recruitment of particular populations of immune cells to the delivery site.

### Macrophages persist in nasal turbinates of INNA-X–treated mice after infection.

**Figure 5:**
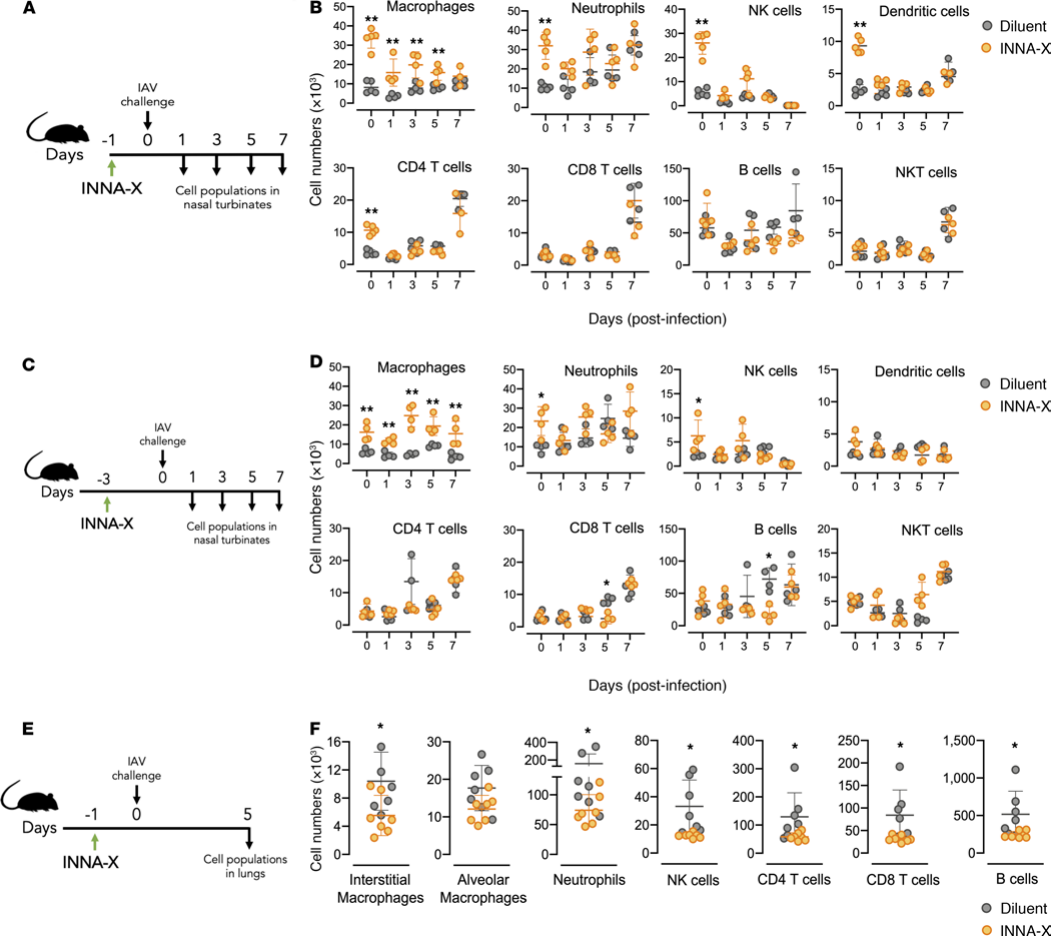
Different cell populations present in the nasal turbinates and lungs after treatment with INNA-X and challenge with influenza virus. (**A** and **B**) Mice (*n* = 3–5/group) were inoculated with 1 nmol of INNA-X or diluent and 1 day later challenged with 500 PFU of Udorn IAV. (**B**) Frequencies of cell populations in the nasal turbinates on 1, 3, 5, and 7 days after challenge were analyzed. (**C** and **D**) Mice (*n* = 4/group) were treated with 1 nmol of INNA-X and 3 days later challenged with virus to examine cell populations present 1, 3, 5, and 7 days after challenge. Cell populations present 24 hours (**B**) or 3 days (**D**) after treatment but prior to challenge with virus are shown in each panel as the day 0 time point. (**E** and **F**) Cell populations in the lungs of inoculated mice (*n* = 7/group) challenged 1 day later were also analyzed at 5 days after infection. Statistical analysis was performed by (**B** and **D**) 2-way ANOVA with a Bonferroni post hoc test or (**F**) a Welch *t* test. *P < 0.01. **P < 0.001.

Mice were treated with INNA-X and 1 day later challenged with influenza virus and ensuing changes in the number of cell populations followed (Figure 5A). Increased numbers of macrophages, neutrophils, NK cells, DCs, and CD4^+^ T cells were detected in animals 1 day after INNA-X treatment (day 0; Figure 5B) compared with diluent treatment, confirming results described above (Figure 4C). After viral challenge, diluent-treated control mice showed a steady rise in neutrophils and elevated levels of DCs, CD4^+^ T cells, CD8^+^ T cells, and NKT cells on day 7 in response to virus challenge. In INNA-X–treated mice, although most cell types displayed similar kinetics to those observed in control animals, macrophage numbers were significantly elevated throughout the course of infection. This indicates that infection 1 day after treatment did not affect INNA-X–dependent recruitment of macrophages. When INNA-X was administered 3 days prior to viral challenge (Figure 5C), macrophage levels were again elevated throughout the course of infection (Figure 5D). In contrast to the nasal turbinates, animals pretreated with INNA-X 1 day prior to viral infection had significantly lower numbers of lung immune cells at 5 days after infection (Figure 5, E and F), correlating with decreased viral titers at this site (Figure 2F) and indicating that protection of the URT against infection reduced the need for immune responses to be elicited in the lower respiratory tract.

### Epithelial cells in addition to macrophages play a role in INNA-X–mediated virus protection.

**Figure 6:**
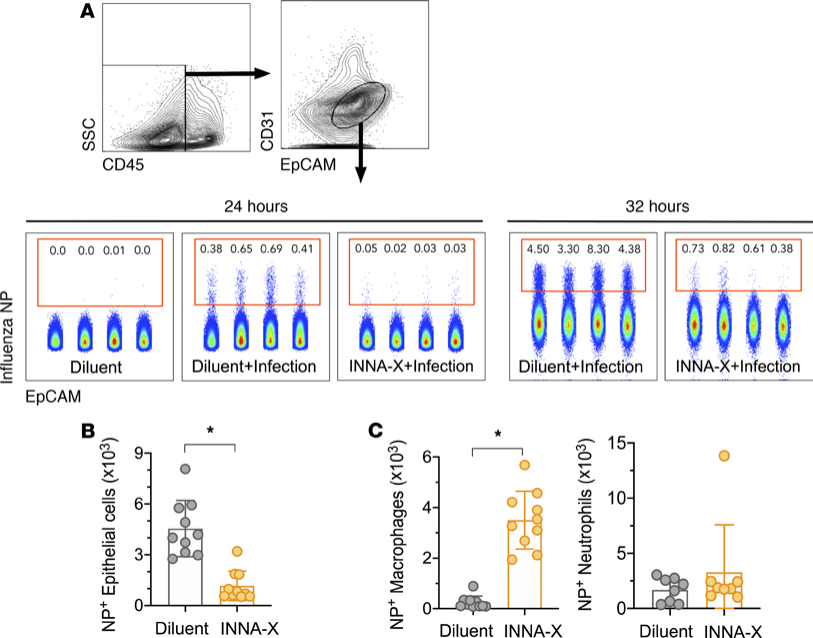
In vivo protection of nasal epithelial cells after viral challenge of mice treated with INNA-X. Mice were inoculated with 1 nmol of INNA-X or diluent and 1 day later challenged with 500 PFU of Udorn IAV. Nasal turbinates were harvested 24 or 32 hours after infection and cell populations analyzed for intracellular influenza virus nucleoprotein (NP) expression. (**A**) Epithelial cells were distinguished by CD45^–^CD31^–^EpCAM^+^ expression. Center panels depict NP^+^-expressing cell populations from individual animals in a representative experiment. (**B**) Bar graphs indicate the total number of NP^+^ epithelial cells, (**C**) NP^+^ macrophages, or NP^+^ neutrophils obtained from 8–10 animals (data pooled from 2 independent experiments). All statistical analysis was performed using a Mann-Whitney *t* test. *P < 0.001.

Because airway epithelial cells can act as a protective barrier against materials, including infectious agents, introduced to the upper airways, we determined whether INNA-X treatment could protect nasal epithelial cells against IAV infection. A clear population of infected cells (intracellular virus nucleoprotein–positive [NP^+^] nasal epithelial [CD45^–^CD31^–^EpCAM^+^]) was detected in diluent-treated control mice 24–32 hours after viral challenge (Figure 6A). Ex vivo analysis of similar cells in INNA-X–pretreated mice revealed significantly fewer NP^+^ cells as early as 24 hours after infection, demonstrating protection of nasal epithelium (Figure 6B), a result that correlated with significantly lower viral titers observed at this site (Figure 2E). In addition, significantly greater numbers of NP^+^ macrophages but not neutrophils were detected in nasal tissues of infected INNA-X–treated mice (Figure 6C) compared with mice treated with diluent and challenged with virus. Macrophages abortively infected by seasonal influenza virus (26, 27) can act as a “sink” for virus (28). This, together with their ability to limit infection through the phagocytosis of apoptotic virus-infected cells (29), may explain the presence of intracellular NP and highlights the contribution of macrophages to INNA-X–mediated protection of the nasal epithelium.

### Dissecting the protective effector roles of nasal epithelial cells and macrophages.

**Figure 7:**
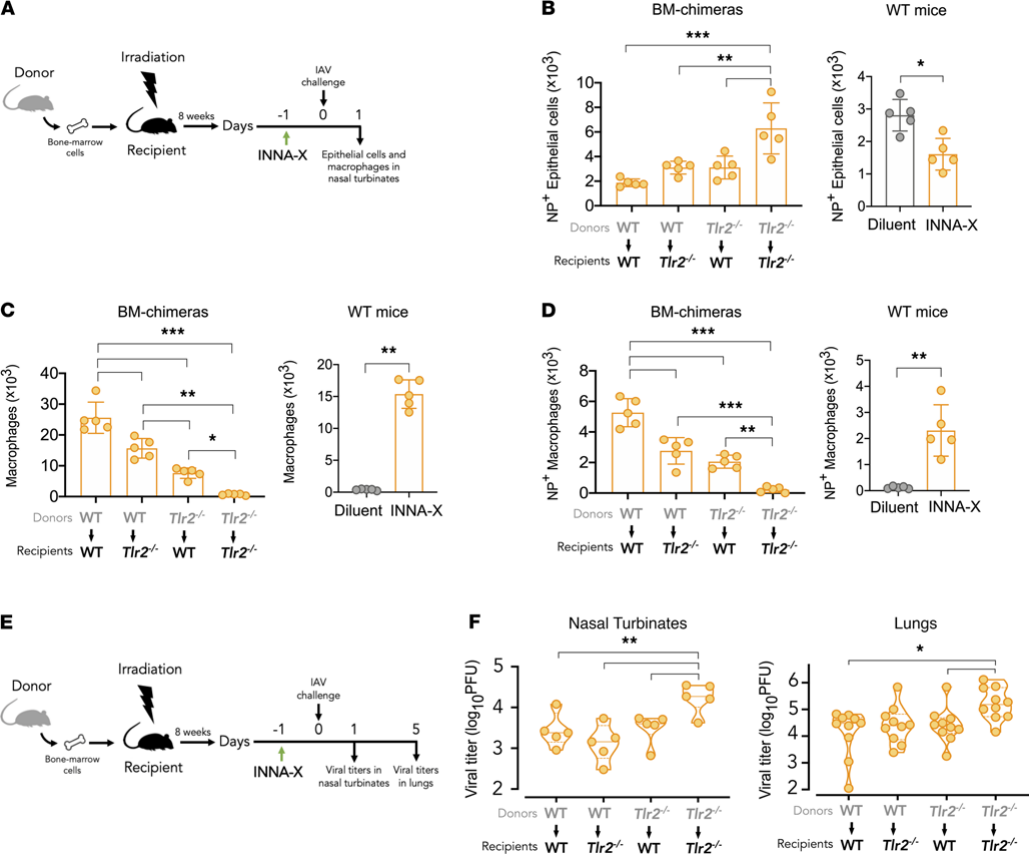
Contribution of immune and nonimmune cells in mediating protection. (**A**) Bone marrow chimeras were established by adoptive transfer of donor bone marrow cells from WT C57BL/6 or *Tlr2^–/–^* mice into irradiated recipient mice (*n* = 5/group). After 8 weeks, animals were inoculated with 1 nmol of INNA-X prior to viral challenge with 500 PFU of Udorn IAV. Separate groups of WT mice were similarly treated with INNA-X and diluent prior to viral challenge. Nasal turbinates were harvested 1 day after challenge and the frequencies of (**B**) NP^+^-expressing CD45^–^CD31^–^EpCAM^+^ epithelial cells, (**C**) total macrophages, and (**D**) NP^+^-expressing macrophages determined. Results representative of 2 experiments conducted independently. (**E** and **F**) Viral titers in the nasal turbinates and lungs were also determined 1 and 5 days after infection, respectively. Statistical analysis of data from bone marrow chimeras (**B**–**D** and **F**) were performed by 1-way ANOVA with Tukey’s post hoc test and data from WT mice analyzed by a Welch *t* test. Comparison of lung viral titers (**F**) analyzed by a Kruskal-Wallis multiple-comparison test. *P < 0.05, **P < 0.01, ***P < 0.001.

We (18) have previously shown that *Tlr2^–/–^* mice are not responsive to INNA-X treatment, and given that both epithelial cells and macrophages likely play a role in mediating antiviral activity in the nasal turbinates of mice treated with INNA-X, we generated chimeras using TLR2-deficient mice (*Tlr2^–/–^*) on a C57BL/6 background to assess the relative roles of these cells. Adoptive transfer of donor cells from WT mice into lethally irradiated *Tlr2^–/–^* recipients (Figure 7A; WT > *Tlr2^–/–^*) provides a mouse model in which only the immune cells from the WT donor will be responsive to activation by INNA-X. In the inverse (*Tlr2^–/–^* > WT) model, epithelial cells but not immune cells will be responsive to INNA-X treatment. We also established chimeras where donor bone marrow cells from either WT or *Tlr2^–/–^* mice were transferred into the same irradiated recipient strains (WT > WT or *Tlr2^–/–^* > *Tlr2^–/–^*) as controls.

When the URTs of chimeric mice were examined for their ability to resist viral challenge, infection of nasal epithelial cells was significantly lower in INNA-X–treated and virus-challenged WT > WT chimeras than in similarly treated and challenged *Tlr2^–/–^* > *Tlr2^–/–^* chimeras (Figure 7B). This trend supported that TLR2 expression is required for mediating the antiviral effect of INNA-X. Protection of WT > WT mice was also associated with higher numbers of nasal macrophages (Figure 7C), including populations expressing intracellular viral NP (Figure 7D), recapitulating our observations in WT mice.

Reduced numbers of NP^+^ epithelial cells were observed in WT > *Tlr2^–/–^* chimeric mice (Figure 7B) compared with *Tlr2^–/–^* > *Tlr2^–/–^* chimeras and were accompanied by increased macrophage levels (Figure 7C). This indicates that WT-derived TLR2-expressing immune cells recruited by INNA-X treatment can act to restrict infection of TLR2^–^ epithelial cells. Similar reduction in nasal infected epithelial cells was also observed in *Tlr2^–/–^* > WT chimeras (Figure 7B). Under the conditions of the experiment, immune cells from *Tlr2^–/–^* mice are not directly stimulated by INNA-X, leading to the conclusion that INNA-X–treated TLR2^+^ epithelial cells can themselves resist infection. This is supported by the lower macrophage numbers found overall (Figure 7C), although NP-expressing macrophages were still present (Figure 7D). Similarly, there were reduced viral titers in the nasal turbinates of WT > *Tlr2^–/–^* and *Tlr2^–/–^* > WT chimeras (Figure 7, E and F) compared with *Tlr2^–/–^* > *Tlr2^–/–^* chimeras. In the case of *Tlr2^–/–^* > WT chimeras, significantly less virus was also detected in the lung (Figure 7F), suggesting that the effects mediated by TLR2-expressing nonimmune cells extends to protection of the lower respiratory tract.

**Figure 8:**
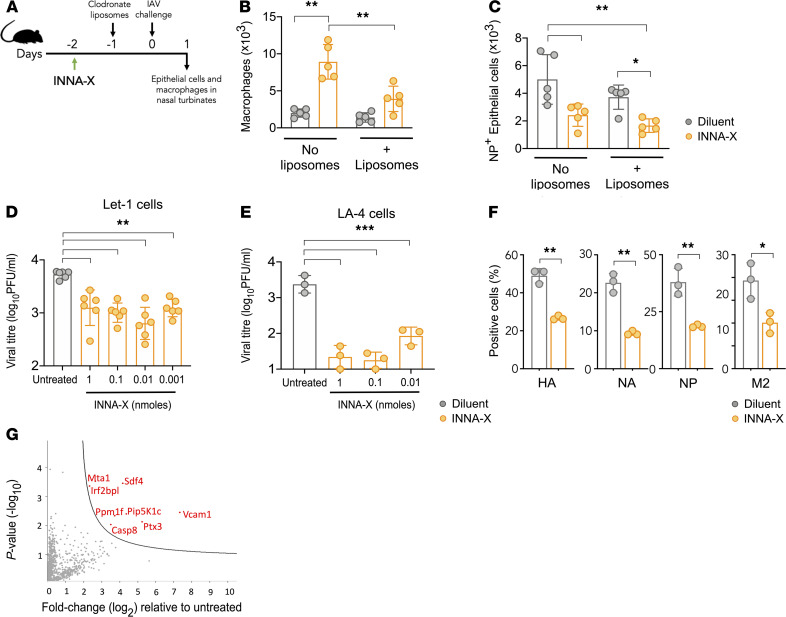
Epithelial cell protection in macrophage-depleted mice and in Let1 and LA-4 cells. (**A**) Mice (*n* = 5/group) were inoculated with 1 nmol of INNA-X or diluent and 1 day later received clodronate liposomes via the i.v. (200 μL) and i.n. route (50 μL) or were left untreated. All mice were challenged the next day with 500 PFU of Udorn IAV and 1 day after viral challenge the numbers of (**B**) macrophages and (**C**) NP^+^ CD45^–^CD31^–^EpCAM^+^ epithelial cells in nasal turbinates were analyzed. (**D** and **E**) 5 × 10^5^ Let1 or 2 × 10^5^ LA-4 cells were incubated in the presence or absence of INNA-X for 24 hours (**D**) or 18 hours (**E**) (*n* = 3–6/group). Cell monolayers were washed prior to challenge with Udorn IAV (MOI of 0.01). Culture supernatants were harvested 24 hours later and assayed to quantitate viral titers. (**F**) LA-4 cells were treated with 1 nmol of INNA-X prior to infection. Cells were harvested 8 hours after infection and stained for surface expression of HA, neuraminidase (NA), matrix-2 (M2) influenza viral proteins, and intracellular expression of NP. (**G**) INNA-X–treated LA-4 cells were lysed and proteins analyzed by reverse-phase liquid chromatography with tandem mass spectrometry (LC-MS/MS) after proteolytic digestion. Sample comparisons were performed using the MaxQuant proteomics platform to determine differentially expressed proteins (red) in INNA-X–treated cells relative to those treated with diluent. Statistical analysis was performed using (**B**–**D** and **E**) 1-way ANOVA with Tukey’s post hoc test and (**F**) Welch *t* test. *P < 0.05, **P < 0.01, ***P < 0.001.

To further clarify the role of macrophages in mediating epithelial cell protection in vivo, mice inoculated with INNA-X were treated with dichloromethylene diphosphonate–loaded (clodronate-loaded) liposomes to deplete macrophages prior to viral challenge (Figure 8A). Administration of liposomes after INNA-X treatment significantly reduced macrophage numbers in the nasal turbinates but did not affect other cell populations examined (Supplemental Figure 3). Upon viral challenge, compared with animals treated with INNA-X only, a significant reduction in nasal turbinate macrophages was detected in INNA-X–treated mice administered liposomes (Figure 8B). In fact, the numbers of macrophages seen in these animals were not significantly different from those observed in animals that received diluent only. However, irrespective of whether macrophages were depleted, treatment with INNA-X resulted in significantly lower numbers of infected epithelial cells (Figure 8C). Taken together, the results of the bone marrow chimera and clodronate depletion experiments suggest that antiviral effects induced by INNA-X are mediated by both macrophage and epithelial cells.

To investigate the direct protective effects of INNA-X on epithelial cells, we determined whether INNA-X could protect murine airway epithelial cells from infection. Infection of murine airway epithelial cell lines Let1 (30) and LA-4 with influenza virus resulted in more than 3.5 log_10_ of infectious virus particles released into the cell supernatant. In contrast, significantly reduced titers of infectious virus were recovered from the supernatants of influenza virus–infected cells pretreated with INNA-X (Figure 8, D and E). These effects also correlate with reduced detection of surface and intracellular viral proteins, including NP, in INNA-X–treated LA-4 cells 8 hours after infection (Figure 8F). Moreover, proteomics analysis of LA-4 cells after INNA-X treatment and prior to infection revealed significant upregulation of mediators involved in apoptosis, chromatin regulation and transcription, leukocyte transendothelial migration, and antiviral activity, most notably pentraxin 3 (PTX3) and caspase-8 (Figure 8G). Taken together, these results indicate that while the influx of macrophages into the nasal turbinates induced by INNA-X treatment can help mediate protection against influenza infection, INNA-X also acts directly on the nasal epithelium to facilitate antiviral resistance.

## Discussion

We have demonstrated that administration of INNA-X, when restricted to the upper airways, resulted in a reduction of viral loads in the nasal epithelia and a decline in viral progression to the lungs. Studies on respiratory pathogens in mice typically utilize administration of material to the respiratory tract in a volume that results in deposition throughout the respiratory tract, which can lead to abnormally exaggerated and pathological sequelae. Clearly, the need to limit inflammatory responses in the lower airways and lungs is mandated, and in the case of infectious agents, this method of inoculation may not mimic natural infection because deposition is initially confined to the URT and precedes dissemination to the lower respiratory tract (6, 31, 32). Experimental delivery of material in large volumes may also mask the contribution of immune effectors and mediators that are present in the URT and normally prevent progression to the lower respiratory tract.

Recently, Hua et al. (33) used small delivery volumes to localize murine coronavirus infection to the nasal tissue and showed that mice are protected against severe pneumonia and death when challenged with unrelated viruses, including IAV. We have shown here that reduction in pulmonary virus can also be achieved using INNA-X, rather than by previous infection, to establish protective innate immune–mediated mechanisms. Exposure of nasal turbinates to INNA-X initiated a local proinflammatory effect through upregulation of cytokines, including IL-6, TNF-α, and IL-1β, exemplifying innate immune responses to influenza infection (8, 34), and the production of chemotactic molecules, including CXCL1 (KC), CCL3 (MIP-1α), and CCL2 (MCP-1), followed by an influx of macrophages, neutrophils, and other lymphocyte populations. These responses mirror those observed in influenza-infected human epithelial cells (35), which activate genes relating to danger sensing and immune cell migration and support the notion that INNA-X preempts and potentiates the antiviral response in epithelial cells.

Of note, macrophages recruited after INNA-X treatment exhibited a predominantly M2 phenotype as characterized by their low expression of MHC class II, CD86, and inducible NOS (iNOS) and high expression of CD206 and Arginase 1 (Arg1) (36, 37) compared with macrophages from diluent-treated animals (Supplemental Figure 4). These results suggest that they are recruited in response to INNA-X–mediated cytokine production to regulate excessive inflammation and limit tissue damage. Their elevated presence at the site of infection nonetheless still contributes to protection given that a proportion of these macrophages expressed intracellular viral protein, indicating that they had been infected abortively (26, 27) and/or that they had phagocytosed apoptotic virus-infected cells (29), serving to limit viral replication (38–40) before too many rounds of amplification took place.

In this regard, our macrophage depletion experiments and the use of bone marrow chimeras appear to suggest that the protective effects observed are predominantly mediated by epithelial cells and further reinforced by their ability to resist infection in the absence of other cell types in vitro. The ability of airway epithelial cells to initiate frontline innate antiviral responses is well known, and while many studies have found the production of IFNs to play a major role (11, 41), our analyses showed no detectable upregulation of gene transcripts for IFNs or significantly increased levels of their products (Supplemental Figure 5) in nasal turbinates after either treatment with INNA-X alone or INNA-X followed by viral challenge when compared with diluent-treated controls. These observations are consistent with our previous findings demonstrating INNA-X treatment of IFN-α receptor–deficient mice (18) are still protected against lethal IAV challenge, thus pointing to other protective mediators at play.

Our analysis of RNA transcripts from nasal turbinates of INNA-X–treated mice detected enrichment of genes involved in the regulation of ERK1/2 signaling cascade, implicating possible involvement of ROS, such as superoxide, hydrogen peroxide, and nitric oxide (14, 42–44). The generation of these molecules forms an important component of host antiviral immune responses in the nasal mucosa and can play a key role in protecting epithelial cells from infection without the requirement for IFN-mediated responses (14). ERK1/2 activity can also be linked to the induction of apoptosis, as evidenced by the detection of caspase-8 activation in INNA-X–treated epithelial cells. In line with this, we have observed the upregulation of genes associated with programmed cell death in INNA-X–treated nasal turbinates, namely those encoding TNF superfamily members, as well as increased expression of Fas (our unpublished observations), thus suggesting treatment primes nasal epithelial cells to undergo apoptosis during the early stages of infection as a countermeasure to limit viral production. In addition, we also detected increased levels of PTX3 produced by INNA-X–treated epithelial cells. PTX3 is an essential mediator of innate resistance to viral pathogens and can be upregulated in the presence of IL-1β and TNF-α (45), cytokines that were present at elevated levels in the nasal turbinates after treatment. Overall, these findings paint a scenario where INNA-X treatment establishes an immunologically alert state in which activated nasal epithelial cells are prepared for a rapid, broadly protective antiviral response as a first line of defense and aided by the recruitment of macrophages to the nasal tissue.

Our findings from this study have clear relevance for the clinical development of INNA-X as an immunomodulator to mobilize rapid innate immunity against respiratory viruses, including influenza and rhinovirus (18–21). We see a role for this protective strategy in prevention of disease in many contexts, such as frontline health workers, nursing homes, travelers, and individuals who have not been vaccinated, or during outbreaks of virus for which there is no vaccine or treatment and even for those who have been recently exposed. Furthermore, it has not escaped our attention that INNA-X treatment can be implemented as a multidosing regimen to extend and provide continuous protection throughout an outbreak. Of note and highly relevant to COVID-19, our recent studies have since demonstrated its protective efficacy against SARS coronavirus 2 in a ferret model (46), establishing proof of concept that this strategy can be applied in the context of rapidly spreading pandemic viruses to protect immunologically naive global populations.

## Methods

### Synthesis of INNA-X.

The synthesis of a pegylated analog of the diacylated lipopeptide Pam_2_Cys, referred to as INNA-X, has been described previously (18). Pam_2_Cys is inherently insoluble and has been rendered soluble by others through addition of the amino acid motif Ser-Lys-Lys-Lys-Lys (SK_4_) (47). Oligo lysine sequences have, however, been shown to be toxic, albeit at high concentration (48), and even to modulate viral infection processes independent of TLR activation (49). We avoided any off-target effects by incorporating polyethylene glycol as a solubilizing agent into the covalent structure of Pam_2_Cys. The final, totally synthetic product contained no detectable lipopolysaccharide (<0.05 endotoxin units/mL) as determined using the Limulus amebocyte lysate assay (Lonza).

### Animals.

Female and male C57BL/6 mice expressing the congenic markers CD45.1 or CD45.2 and *Tlr2^–/–^* mice aged 6–9 weeks were used in this study and were bred and maintained in the Biological Research Facility in the Department of Microbiology and Immunology at the University of Melbourne.

### Treatment with INNA-X and viral challenge.

Mice were anesthetized with isoflurane and inoculated by i.n. instillation with INNA-X, A/Udorn/307/72 (H3N2) IAV (Udorn IAV), or diluent. The inoculum was confined to the URT by limiting the volume administered dropwise onto the nares to 10–15 μL; dissemination into the entire respiratory tract was achieved by using an inoculation volume of 40–50 μL. Udorn IAV was propagated in the allantoic cavity of 10-day-old embryonated hens’ eggs; eggs were inoculated with approximately 10^3^ PFU of virus in 0.1 mL of saline. After 2 days of incubation at 35°C, eggs were chilled at 4°C, and allantoic fluid was harvested, clarified by centrifugation at 720*g* for 5 minutes, and stored at –80°C until used.

### Localization of inoculum determined using Evans blue dye.

Anesthetized mice were i.n. inoculated with 10, 15, 20, or 50 μL of PBS containing 0.125% (w/v) Evans blue dye (MilliporeSigma). After 5 minutes, mice were killed and respiratory tissues (nasal turbinates, trachea, and lung) and stomach tissues harvested. Tissues were rinsed in PBS and homogenized in 1 mL of PBS using a probe homogenizer. Protein was precipitated by addition of 1 mL 100% trichloroacetic acid and centrifugation at 1500*g* for 1 hour. The optical extinction at 620 nm of the supernatants was compared with a standard curve using a Titertek Multiskan plate reader. Concentrations of dye in tissues were calculated by interpolation from the standard curve using optical density as a function of dye concentration.

### Harvesting of tissue and preparation of sera.

Viral titers and cytokines were measured in the nasal turbinates and lungs harvested from mice and collected into 1.5 mL of RPMI-1640 medium (MilliporeSigma) containing antibiotics (100 μg/mL penicillin, 180 μg/mL streptomycin, and 24 μg/mL gentamicin). All samples were kept on ice until processed. Tissues were homogenized and clarified supernatants, obtained by centrifugation, were stored at –80°C for subsequent study. Blood, approximately 200 μL, was obtained from mice by venipuncture of the submandibular vein. Blood was allowed to clot at 4°C overnight and serum separated by centrifugation at 6000*g* in a microfuge for 5 minutes and stored at –80°C.

### Determination of viral titers.

Titers of infectious virus were determined by plaque assay on confluent monolayers of Madin Darby canine kidney cells (MDCK cells; tissue culture cell repository; The Department of Microbiology and Immunology, The University of Melbourne, at the Peter Doherty Institute). Six-well tissue culture plates were seeded with 1.2 × 10^6^ MDCK cells per well in 3 mL of RP10 (RPMI-1640 medium supplemented with 10% [v/v] heat-inactivated FCS, 260 μg/mL glutamine, 200 μg/mL sodium pyruvate, and the antibiotics indicated above). After overnight incubation at 37°C in 5% CO_2_, confluent monolayers were washed with RPMI containing antibiotics. Test supernatants serially diluted in RPMI containing antibiotics were added to wells of monolayers and incubated at 37°C in 5% CO_2_ for 45 minutes. Monolayers were then overlaid with 3 mL of Leibovitz L15 medium (pH 6.8) containing 0.9% agarose and 2 ug/mL trypsin-TPCK supplemented with antibiotics. Plates were incubated for 3 days at 37°C in 5% CO_2_ and PFUs were counted without staining. All assays were carried out in duplicate.

### RNA extraction from nasal turbinates.

RNA was extracted from nasal turbinates using a RNeasy Mini Kit (Qiagen). Briefly, turbinates were placed in 600 μL of Qiagen RLT buffer containing 1% (v/v) β-mercaptoethanol (Calbiochem) and vortexed for 30 seconds. The lysate was homogenized using QIAshredder columns (Qiagen) before addition of an equal volume of 70% (v/v) ethanol. The RNA was then isolated according to the manufacturer’s instructions. RNA quality and concentration were determined using a LabChip microfluidic capillary electrophoresis system (PerkinElmer).

### Quantitation of gene expression by NanoString analysis.

RNA (50 ng per sample) was hybridized with reporter and capture probe sets from the nCounter mouse immunology panel (XT_PGX_MmV1_Immunology; NanoString Technologies) at 65°C for 16 hours in a Bio-Rad thermocycler. Targets were annealed to nCounter cartridges using the nCounter prep station and were imaged using the nCounter digital analyzer set to 280 fields of view (NanoString Technologies).

### Analysis of differential gene expression.

Quality control of nCounter data and export of raw count data were performed using nSolver version 4.0 software. Differential expression analyses were performed online using the Degust version 3.1.0 tool (50) using the Voom/Limma package. Raw counts were used as input with a minimum count threshold of 10 applied (approximately 2 standard deviations above the mean count of negative control probes). Differential expression was defined as log_2_ fold change compared with PBS-treated controls of 1.00 or more or –1.00 or less (i.e., at least 2-fold), with an FDR less than 0.05. Volcano plots were generated using the EnhancedVolcano package (51) in R version 3.5.1 and heatmaps generated using Heatmapper (52) with genes clustered by Euclidian distance. Gene ontology terms were identified using Database for Annotation, Visualization and Integrated Discovery version 6.8 (53) and gene functional annotation tools.

### Characterization of cell populations in nasal turbinates.

Each turbinate was incubated for 30 minutes at 37°C in a solution of RPMI-1640 containing 1 mg/mL collagenase A and 0.5 mg/mL DNase I (Roche Diagnostics) before being pressed through a metal sieve to collect single-cell suspensions. Red blood cells were lysed by treatment with 0.15 M NH_4_Cl and 17 mM Tris-HCl at pH 7.2 for 5 minutes at 37°C. Immune cells were detected using combinations of the fluorochrome-conjugated anti-mouse antibodies for 30 minutes at 4°C. The antibodies used were FITC-NK1.1 (clone PK136), FITC-iNOS (6/iNOS/NOS Type II [RUO]), PE-SiglecF (clone E50-2440), PerCP-Cy5.5-CD8 (clone 53-6.7), PE/Cy7-CD3 (clone 17A2), and AF647-CD64 (clone X54-5/7.1) from BD Biosciences; FITC-CD11b (clone M1/70), Pacific Blue-CD11b (clone M1/70), PE-CD4 (clone GK1.5), PE-CD206 (clone MMR), PerCP-Cy5.5-GR1 (clone RB6-8C5), PerCP-Cy5.5-Ly6G (clone 1A8), APC/Cy7-F480 (clone BM8), and APC-B220 (clone RA3-6B2) from BioLegend; PE-CD16/32 (clone 93), PE/Cy7-CD11c (clone N418), AF700-Arg1 (clone A1exF5), and AF700-CD45 (clone 30-F11) from eBioscience, Thermo Fisher Scientific. CD45^+^ cell populations were classified as follows: interstitial macrophages, Ly6G^–^F480^+^SiglecF^–^CD64^+^CD11b^+^CD11c^+^; alveolar macrophages, Ly6G^–^F480^+^SiglecF^+^CD64^+^CD11b^–^CD11c^+^; neutrophils, F480^–^SiglecF^–^CD11b^hi^GR1/Ly6G^hi^; DCs, F480^–^GR1^–^CD11b^hi^ CD11c^hi^; CD4^+^ T cells, CD3^+^CD4^+^; CD8^+^ T cells, CD3^+^CD8^+^; B cells, CD3^–^B220^+^; NK cells, CD3^–^NK1.1^+^CD16^+^CD32^+^; and NKT cells, CD3^+^NK1.1^+^.

Intracellular detection of Arg1 and iNOS in macrophages was performed after surface marker staining. Cells were fixed, permeabilized, and incubated with fluorochrome-conjugated antibodies using a Cytofix/Cytoperm Fixation/Permeabilization Kit (BD Biosciences) according to the manufacturer’s instructions. Cells were washed extensively, and analysis was carried out using a BD Biosciences LSR II or Canto II flow cytometer and data analyzed using FlowJo software (Tree Star, Inc.).

Epithelial cells were identified by staining with BV421-CD31 (clone 390) and PerCP-CD45 (clone 30-F11) from BD Biosciences and APC-Cy7-EpCAM (clone G8.8) from BioLegend. Detection of intracellular influenza virus NP was performed by permeabilization and fixation of cells using the Foxp3 Transcription Factor Staining Buffer Set (Thermo Fisher Scientific) followed by staining with FITC-conjugated anti-NP antibody (clone 1331, Genetex) according to the manufacturers’ instructions.

### Analysis of cytokine levels.

IFN-γ, IL-2, IL-4, TNF-α, IL-10, IL-6, KC, MCP-1, RANTES, IL-12/IL-23p40, and IL-17A present in homogenate and serum samples were measured using the BD Cytometric Bead Array (CBA) flex kit according to the manufacturer’s instructions, with the exception that a total of 0.15 μL of each capture bead suspension and 0.15 μL of phycoerythrin detection reagent was used in each 50 μL sample. For the detection of cytokines not covered in the CBA kit, a LEGENDplex custom panel or a mouse antivirus response mix and match subpanel was used (BioLegend). Samples were analyzed using a BD FACSCanto II flow cytometer, and data were analyzed using FCAP Array software (BD Biosciences) or LEGENDplex version 7.1 software (VigeneTech Inc.). Where data fell below the lower limit of quantitation, data points were displayed at the limit of quantitation for visual and statistical analysis. IFN-λ levels in nasal turbinate samples were measured using a mouse IFN-λ2/3 (IL-28A/B) ELISA kit according to the manufacturer’s instructions (PBL Assay Science).

### Generation of bone marrow chimeras.

Bone marrow chimeras were established using a method previously described (54). Briefly, CD45.1-expressing C57BL/6 WT and *Tlr2^–/–^* mice were lethally irradiated with 2 doses of 5.5 Gy 3 hours apart and reconstituted with 5 × 10^6^ T cell–depleted bone marrow cells from CD45.2- or CD45.1-expressing WT or *Tlr2^–/–^* mice as previously described. Donor and recipient cells were discriminated by isoform expression in vivo. Mice were depleted of radioresistant T cells on the following day by i.p. injection with anti-Thy1 antibody (clone T24; in the form of 100 µL of cell culture supernatant manufactured in-house). Animals were maintained on antibiotics (neomycin and polymyxin B, MilliporeSigma) contained in drinking water for 6 weeks. Chimeric mice were allowed to reconstitute for at least 8 weeks, by which time they reached an average reconstitution of 93.5%.

### In vitro infection of lung epithelial cells.

The adherent lung alveolar epithelial type 1–like cell line, Let1, was cultured in DMEM (Invitrogen, Thermo Fisher Scientific) supplemented with 10% (v/v) heat-inactivated FCS, 260 μg/mL glutamine, 200 μg/mL sodium pyruvate, and antibiotics (100 μg/mL penicillin, 180 μg/mL streptomycin, and 24 μg/mL gentamicin). LA-4 mouse lung epithelial cells were cultured in Kaighn’s modification of Ham’s F12 medium (Gibco, Thermo Fisher Scientific) supplemented with 10% (v/v) heat-inactivated FCS, 4 mM l-glutamine, nonessential amino acids (MilliporeSigma), 100 IU of penicillin, 10 μg of streptomycin/mL, and 50 μM of 2-mercaptoethanol (Gibco, Thermo Fisher Scientific).

Monolayers were grown to 80%–90% confluence before harvesting using trypsin versine and distributed in 12-well plates at a concentration of 5 × 10^5^ cells (Let1) or 2 × 10^5^ (LA-4) per well in a volume of 1 mL. After establishment of monolayers, medium was replaced with fresh medium containing INNA-X or left untreated and incubated at 37°C in 5% CO_2_ for 24 hours. Culture supernatants were then aspirated, and cells were washed prior to the addition of Udorn IAV in 100 μL (Let1) or 200 μL (LA-4) at an MOI of 0.01. Cells were incubated for 1 hour, and then virus-containing medium was aspirated and replaced with fresh media or serum-free media with 0.5 μg/mL of TPCK-treated trypsin (Worthington Biochemical Corporation) for LA-4 cells and incubated for a further 24 hours. Viral titers were determined in harvested cell culture supernatants by plaque titration on MDCK cells.

To analyze viral protein expression in LA-4 cells, cells were infected with X-31 IAV as described above. At 8 hours after infection, infected cells were harvested with a scraper in PBS containing 1 mM EDTA. Surface expression of viral proteins was detected using a goat anti-H3 HA polyclonal antibody (NR-3118, BEI Resources), goat anti-NA polyclonal antibody (NR-3137, BEI Resources), and mouse anti-M2 (clone 14C2, Abcam) in conjunction with anti-goat Ig-Alexa 647 (Invitrogen, Thermo Fisher Scientific, A21447) or anti-mouse Ig-Alexa 647 conjugates (Invitrogen, Thermo Fisher Scientific, A31571), respectively, and analyzed by flow cytometry. Detection of intracellular influenza NP was performed by permeabilization (0.5% Triton X-100 in PBS) and fixation of cells (0.25% Triton X-100, 1 mM EDTA, and 1% FCS in PBS) followed by staining with FITC-conjugated anti-NP antibody (clone 1331, Genetex) prior to flow cytometric analysis.

### Proteomics analysis of lung epithelial cells.

INNA-X or diluent-treated LA-4 cells were lysed, and proteins were reduced and alkylated in a guanidium chloride lysis buffer (55). Protein quantitation by BCA assay and precipitation were followed by a urea-based trypsin proteolytic digest (56). Samples were desalted using Empore C18 resin (57), and label-free quantitation analysis was performed using reverse-phase LC-MS/MS using an Orbitrap Elite (Thermo Fisher Scientific). Sample comparisons were performed using the MaxLFQ package in the MaxQuant proteomics platform (58) v1.5.3.30. The mass spectrometry proteomics data have been deposited to the ProteomeXchange Consortium via the PRIDE (PubMed ID: 30395289) partner repository with the data set identifier PXD023041.

### Statistics.

All data were tested for normality using a Shapiro-Wilk test to inform the appropriate statistical analysis. For nonparametric analysis between 2 groups, an unpaired 2-tailed Mann-Whitney *t* test was used; for analysis between 3 or more groups, the Holm-Sidak or Kruskal-Wallis test for multiple comparisons was used. For parametric analysis between 2 groups, an unpaired 2-tailed Welch *t* test was used. When 3 or more data sets were compared, 1-way ANOVA with Tukey’s post hoc test was used, but when 3 or more groups with 2 or more time points were compared, a 2-way ANOVA with a Bonferroni post hoc test was used. A *P* value less than 0.05 was considered statistically significant. Statistical analyses were performed using GraphPad Prism, version 7.0. For the proteomic comparisons, Student’s *t* tests were used to compare treatment groups and Benjamini Hochberg multiple hypothesis correction applied using an FDR of 0.05.

### Study approval.

All animal experimentation was conducted in accordance with institutional regulations following review and approval (permit numbers 1914894 and 1513638) by the University of Melbourne Animal Ethics Committee.

## Author contributions

CW, GD, HAQ, SLL, DCJ, and BYC were responsible for the design and conduct of experiments, data analysis, interpretation of results, and drafting of this manuscript. The order of co–first authors reflects the time invested by each author. AB, MC, XJ, and KH performed experiments. WZ, JG, NES, NWB, PCR, and KK provided assistance in the analysis and interpretation of results. LEB provided assistance in the interpretation of results and editing. FM and CD were involved in project management and experimental design. All authors were involved in the editing of this manuscript.

## Supplementary Material

Supplemental data

## Version 1 02/09/2021

In-Press Preview

## Version 2 03/08/2021

Electronic publication
