# Supplementary material for: TLR2-mediated activation of innate responses in the upper airways confers antiviral protection of the lungs
Source: JCI Insight. 2021 Mar 8;6(5):e140267. doi: 10.1172/jci.insight.140267 (PMC8021123; doi:10.1172/jci.insight.140267)
Supplement: Supplemental data [file jciinsight-6-140267-s085.pdf]

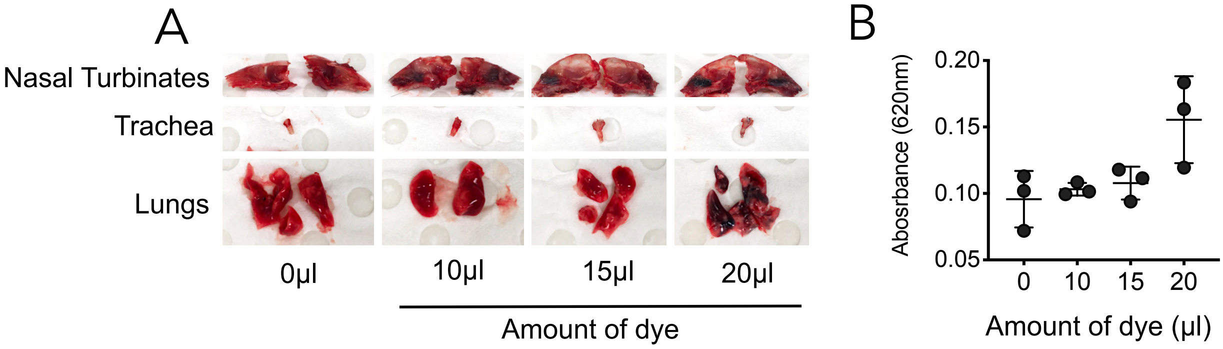

Supplementary Figure 1. Administration of increasing volumes of Evans blue dye and distribution within the respiratory tract. Increasing volumes of PBS containing 0.125% Evans blue dye were administered intranasally to anaesthetised mice (n=3/group). **(A)** Mice were killed by CO_2 asphyxiation 5 minutes after Evans blue administration and nasal turbinates, trachea and lungs harvested for visual inspection. **(B)** Evans blue in mouse lungs was quantified by reading the absorbance (at 620nm) of acid precipitated, clarified lung supernatants.

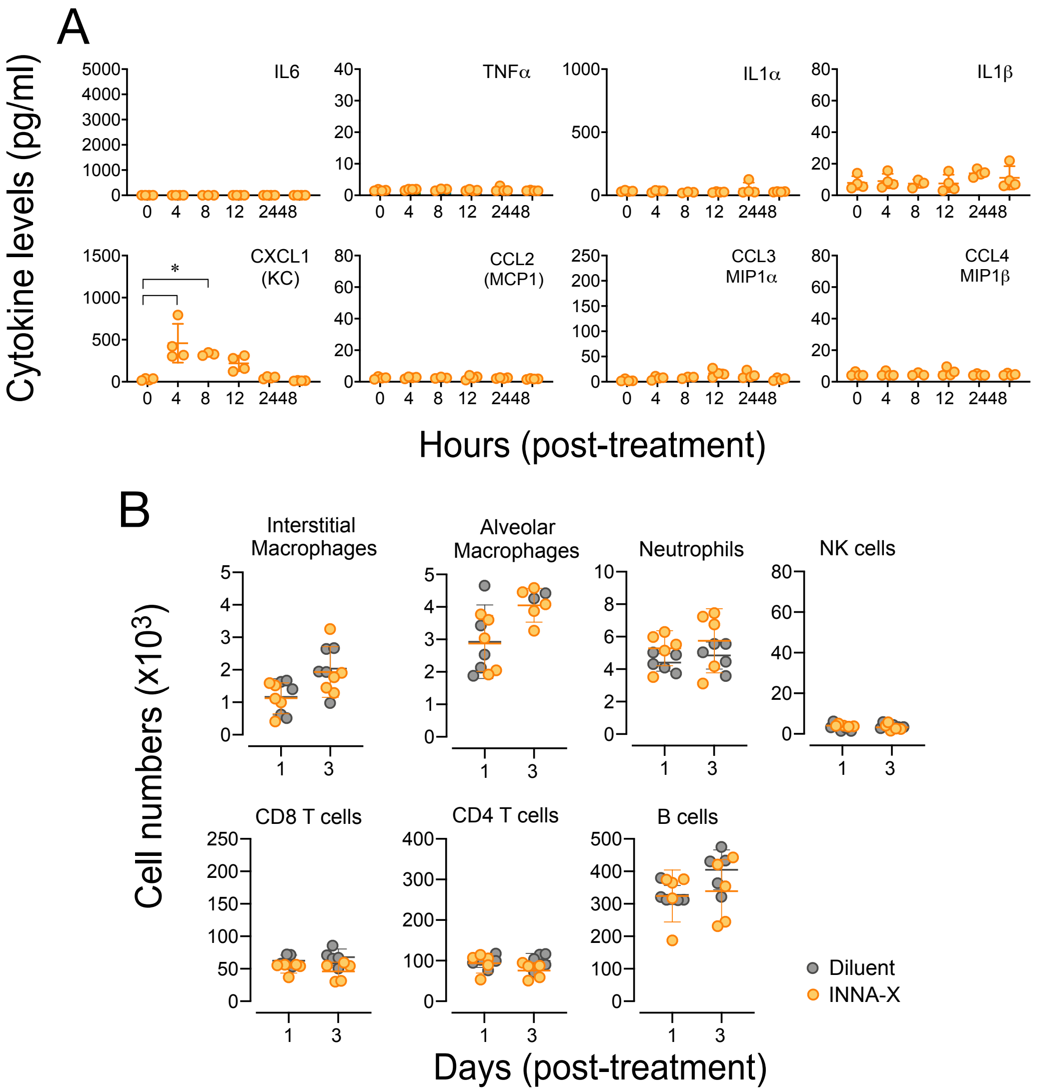

Supplementary Figure 2. Cytokines and cell populations in the lungs following INNA-X treatment. Mice (n=4/group) were inoculated with 1nmole of INNA-X or diluent only and at various time points, (A) cytokine levels in nasal turbinates were analysed in a multiplex bead array assay. (B) Lungs from mice (n=5/group) treated similarly were also harvested 1 and 3 days later and cell populations analysed by flow cytometry. Statistical analysis was performed by (A) one-way ANOVA with a Tukey post-hoc test and (B) two-away ANOVA with a Bonferroni post-hoc test. **p<0.05.*

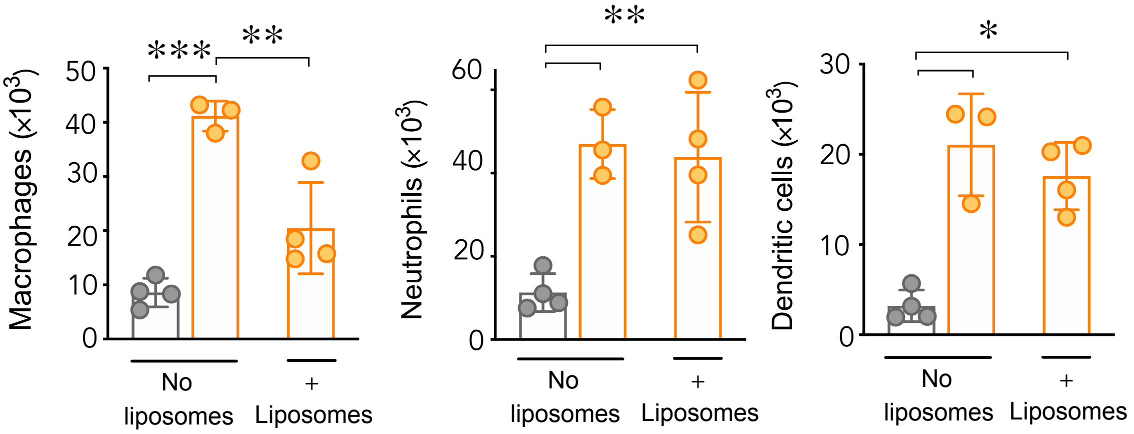

Supplementary Figure 3. Clodronate administration results in selective depletion of macrophages
Mice (n=3/group) were inoculated with 1nmole of INNA-X or diluent and 1 day later received clodronate liposomes via the intravenous (200µl) and intranasal route (50µl) or were left untreated. Nasal turbinates were harvested 1 day later and cell populations analysed by flow cytometry. Statistical analysis was performed by one-way ANOVA with a Tukey post-hoc test. *$^{*}p<0.05$, $^{**}p<0.01$, $^{***}p<0.001$.*

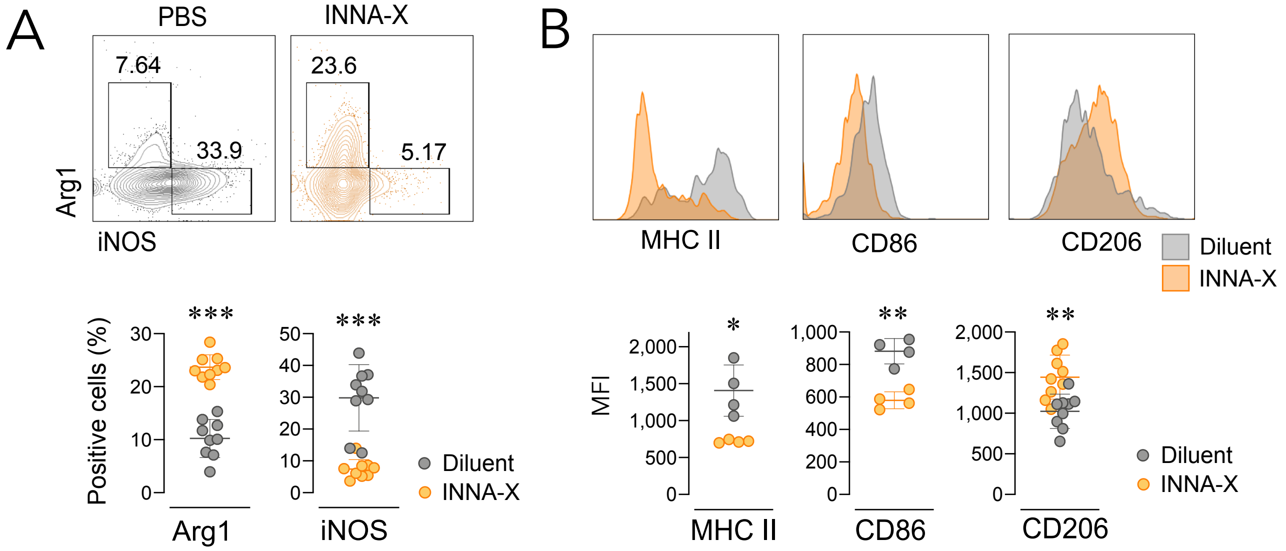

Supplementary Figure 4. M1 and M2 phenotype of nasal turbinate macrophages following INNA-X treatment. Mice (n=4-9/group) were inoculated with 1nmole of INNA-X or diluent only. Nasal turbinates were harvested 1 day later and F480^{+} macrophages analysed for expression of (A) intracellular (Arg-1 and iNOS). The results depict (top) a representative dot plot from a single animal and (bottom) percentage of positive cells for each marker within each group. (B) Cells were also analysed for surface expression of MHC II, CD86 and CD206 with (top) a representative histogram from a single animal and (bottom) mean fluorescence intensity (MFI) of each marker within each group. Statistical analysis was performed using a Welch t-test. **p<0.05, **p<0.01, ***p<0.001.*

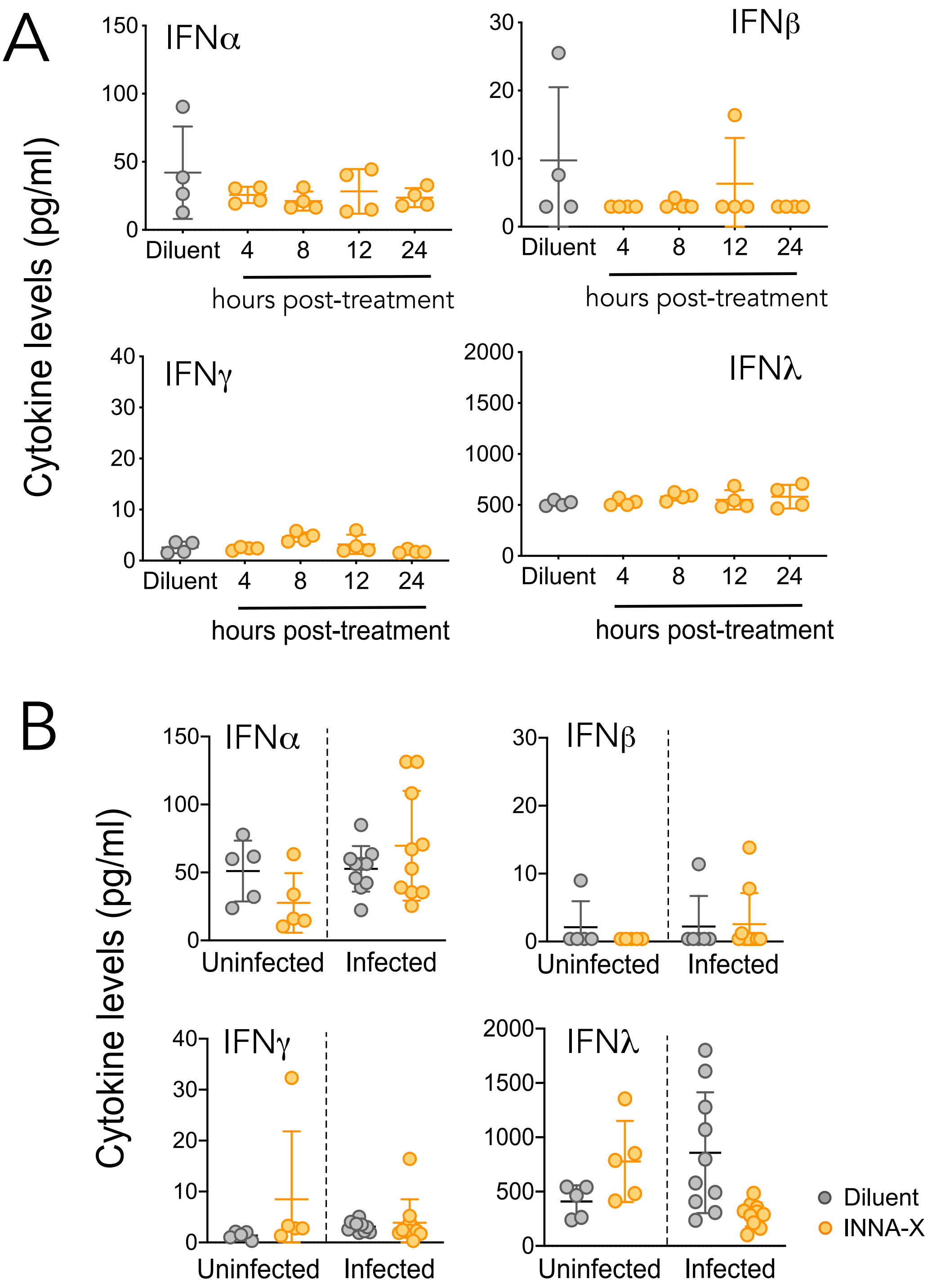

Supplementary Figure 5. IFN levels in the nasal turbinate following INNA-X treatment and virus challenge. **(A)** Mice (n=4/group) were inoculated in the URT with 1nmole of INNA-X and 4, 8, 12 or 24 hours later, IFN levels in the nasal turbinates were measured. Levels of IFN were also determined in mice treated 24-hours prior with diluent. **(B)** IFN levels were measured in the nasal turbinates of mice treated 1 day earlier with diluent or INNA-X but were not challenged with virus. Separate groups of identically treated animals (n=5/group) were challenged with 500pfu of Udorn IAV 1 day after inoculation with INNA-X and IFN-levels present in nasal turbinates measured at 1 day after viral challenge.
